# The top 100 most-cited articles on exercise therapy for sarcopenia: A bibliometric analysis

**DOI:** 10.3389/fmed.2022.961318

**Published:** 2022-08-12

**Authors:** Zhao-jing Guo, Wen-qing Xie, Zi-jun Cai, Yue-yao Zhang, Yi-lan Ding, Shinen Naranmandakh, Yu-sheng Li, Wen-feng Xiao

**Affiliations:** ^1^Deparment of Orthopedics, Xiangya Hospital, Central South University, Changsha, China; ^2^Xiangya School of Medicine, Central South University, Changsha, China; ^3^National Clinical Research Center for Geriatric Disorders, Xiangya Hospital, Central South University, Changsha, China; ^4^School of Arts and Sciences, National University of Mongolia, Ulaanbaatar, Mongolia

**Keywords:** bibliometrics, sarcopenia, exercise, therapy, web of science

## Abstract

**Objective:**

Vast quantities of literature regarding the applications of exercise therapy for sarcopenia have been published. The main objective of this study is to determine the top 100 most-cited articles and analyze their bibliometric characteristics.

**Design:**

This study reports a bibliometric analysis *via* a systematic search of the academic literature regarding the applications of exercise therapy for sarcopenia.

**Methods:**

All databases in the Web of Science were searched with the following strategy: term search (TS) = (exercise^*^ OR training OR “physical activit^*^”) AND TS = (sarcopenia) on 25 February 2022. The results were presented in descending order by their total citations. The list of the top 100 articles was finally determined by negotiation of two independent researchers.

**Results:**

The top 100 articles were published between 1993 and 2020. More than half of the articles (*n* = 54) were published during the decade 2006–2015. Total citations of the top 100 articles ranged from 155 to 1,131 with a median of 211.5. The average of annual citations was constantly increasing with year (*P* < 0.05). The most studied exercise therapy is strength/resistance training, with about 71% articles had discussed about it. The top 100 articles were from 54 different journals, and the *Journal of Applied Physiology* was the journal that contributed the most articles (*n* = 8). A total of 75 different first corresponding authors from 15 countries made contributions to the top 100 list. Luc J.C. van Loon from the Maastricht University in the Netherlands published the most articles (*n* = 5) as the first corresponding author. Most articles (87%) were from North America (58%) and Europe (29%), while the United States as a country contributed over half of the articles (51%).

**Conclusion:**

Our study determined the top 100 most-cited articles on exercise therapy for sarcopenia and analyzed their bibliometric characteristics, which may provide a recommended list for researchers in this field and pave the way for further research.

## Introduction

Sarcopenia is a progressive and generalized skeletal muscle disorder involving the accelerated loss of muscle mass, strength, and functions (1). The decline in muscle quality, which refers to strength per unit muscle cross-sectional area (CSA) or strength per unit muscle mass, appears inevitably with aging, and what is more significant is that such decline is closely associated with physical disabilities (2). As an age-driven phenomenon, if sarcopenia progresses beyond a threshold of functional requirements, such disorder could cause grave physiological and clinical consequences, for instance, increased adverse outcomes, such as falls, fractures, frailty, and mortality (3–9). Sarcopenia can also result in poorer postoperative outcomes and prognosis of various cancer, significantly increasing the risk of metabolic syndrome (10). Patients with sarcopenia showed a higher likelihood of unfavorable functional outcomes 90 days after acute ischemic stroke or transient ischemic attack (11).

Since 2016, sarcopenia has been recognized as a disease, code ICD-10-CM (M62.84), by the World Health Organization (WHO)'s International Statistical Classification of Diseases and Related Health Problems (ICD) (12). In this era of rapid global population aging, the number of older adults with sarcopenia is continuing to be higher. At present, the prevalence data of sarcopenia vary greatly among different regions and races. The prevalence of sarcopenia in older adults in Asia is approximately 5.5–25.7% (13), while in European communities, it is 1–29% (14). In the United States, the prevalence of sarcopenia ranges from 2.5 to 27.2% in women and from 3.1 to 20.4% in men (15), while in Canada, the data became 17.8 and 38.9% in women and men (16), respectively. In Turkey, Bahat et al. (17) investigated a total of 242 community-dwelling outpatients with a mean age of 79.4 ± 5.7 years and found that the prevalence of sarcopenia was 0.8%. For healthy aging, countermeasures to treat the disease need to be strongly emphasized (18). As a traditional treatment, exercise therapy has achieved great success in reversing the age-related changes caused by sarcopenia (19). According to the US Centers for Disease Control and Prevention (CDC), “exercise” was defined as “A subcategory of physical activity that is planned, structured, repetitive, and purposive in the sense that the improvement or maintenance of one or more components of physical fitness is the objective” (20). A range of exercise interventions, including resistance, endurance, as well as other power and functional training, have been used. In particular, progressive resistance exercise training (PRT), in which participants exercise against an increasing external load, has been shown to have positive effects on strength and physical function (21).

Vast quantities of literature regarding the applications of exercise therapy for sarcopenia have been published, describing the practical strategies, effects, or mechanisms as for certain therapy. To the best of our knowledge, there are still no bibliometric articles on the theme of exercise therapy for sarcopenia. To clearly recognize the growing trend and carry out further research in this area, it is necessary to intensively analyze the studies for their influence and contributions in this field (22), and bibliometric analysis is a suitable method to meet the requirements.

## Methods

### Searching strategy

**Figure 1:**
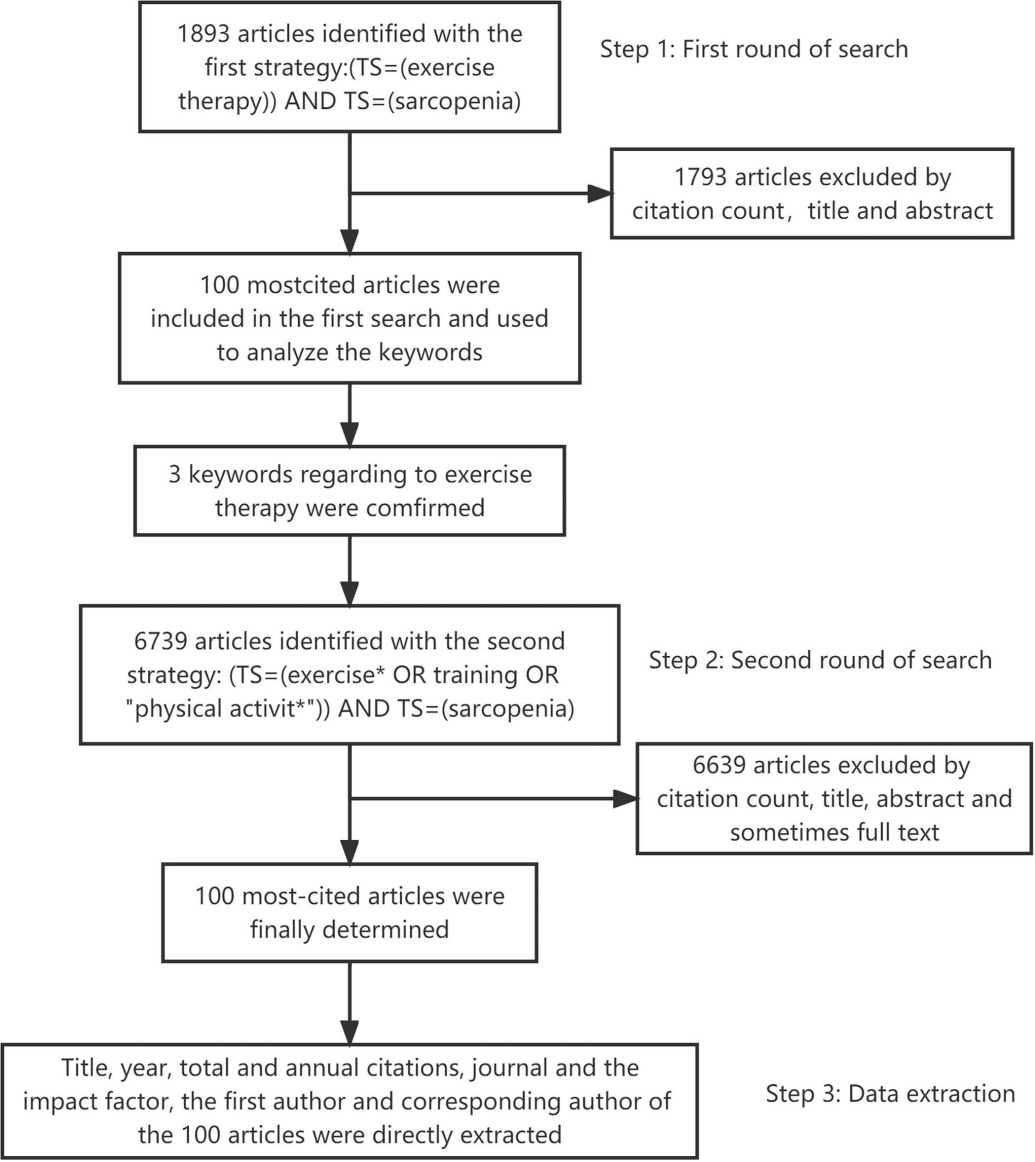
Flowchart showing the approach to identifying the top 100 most-cited articles.

The top 100 most-cited articles were retrieved from the database Web of Science (Web of Science core database, KIC-Korean Journal Database, Medline, Russian Science Citation Index, and SciELO Citation Index) on 25 February 2022. Using three main steps, we identified the top 100 most-cited articles and obtained their information as shown in Figure 1. To get a more comprehensive result, we carried out two rounds of search. In the first round of search, we used the strategy [TS = (exercise therapy)] AND TS = (sarcopenia) and identified 100 most-cited articles from 1,893 results by citation count, title, and abstract. By analyzing their keywords, we finally decided to use three different keywords associated with exercise to represent exercise therapy. Afterward, we started the second round of search on the database with the following strategy: [TS= (exercise^*^ OR training OR “physical activit^*^”)] AND TS=(sarcopenia), with no limitations on published year, languages, and document types. Results were placed in descending order according to their times cited in the Web of Science database. For articles with the same total citation, recent articles were ranked higher because they had less chance to be cited. One of the authors (ZJG) screened the title, abstract, and sometimes full text to identify the qualified articles. We included articles that studied or reviewed the use of exercise therapy in sarcopenia. Articles that had no relative content about this topic or only focused on other therapies were excluded. When certain articles are hard to decide whether to be included, we referred to another author (WQX), and the two authors finally achieved agreement on the list of top 100 most-cited articles.

### Data extraction

The list of top 100 most-cited articles on exercise therapy for sarcopenia was extracted to Microsoft Excel 2019. Title, total citations, annual citations, journal of publication, year of publication, impact factors of the journal, the first author, and corresponding author were directly extracted from the search result. Article type was defined as an original article or a review based on the search result and subsequent check. The research focus was determined by researcher Zhaojing Guo by screening the abstract, full text, and keywords of the 100 articles. Country and institute were recorded according to the first corresponding author's information for each article. Journal impact factors were queried from 2020 Incites Journal Citation Reports.

### Statistical analysis

The annual citation was calculated by the formula: (total citations)/(2022- “publication year” + 1). Statistical analyses were performed using SPSS. The correlation analysis was performed using Pearson's correlation analysis. A *P-*value of < 0.05 was considered statistically significant. Quantitative data were presented as median [first quartile (Q1), third quartile (Q3)]. Both the bar charts and the pie charts in the manuscript were drawn by Microsoft Excel 2019.

### Patient and public involvement

Patients and/or the public were not involved in the design, conduct, reporting, or dissemination plans of this research.

## Results

We retrieved the qualified top 100 most-cited articles with regard to exercise therapy for sarcopenia from a total of 6,739 search results. The 100 articles are listed in Supplement Table 1 according to the descending sequence by the total citations as the primary keywords and the annual citations as the secondary keywords.

### Year of publication

**Figure 2:**
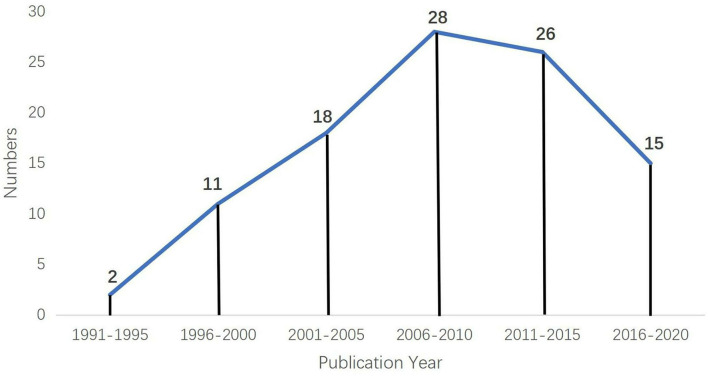
Distribution of the top 100 articles in each 5-year interval.

The distribution of the top 100 articles in each 5-year interval is shown in Figure 1. The top 100 most-cited articles were published between 1993 and 2020, and the oldest publications were “Changes in skeletal muscle with aging: effects of exercise training” published in *Exercise and Sport Sciences Reviews* by Marc A. Rogers and William J. Evans, and “Sarcopenia and Age-Related Changes in Body Composition and Functional Capacity” published in *The Journal of Nutrition* by William J. Evans and Wayne W. Campbell, both published in 1993. Figure 2 showed the number of articles published in each 5-year interval. There were only two articles published before 1995, and both were published in 1993. However, more than 10 were published after 1995 for each interval. More than half of the articles (*n* = 54) were published in the decade 2006–2015. The largest number of articles published in a single 5-year interval (i.e., 2006–2010) was 28.

### Citations

Total citations of the top 100 cited articles were between 155 and 1,131. The median of the total citations was 211.5 (183, 309). The sum of total citations of the top 10 articles accounted for more than a quarter (26.5%, *n* = 7472) of the sum of the top 100 articles. Annual citations of the top 100 articles were between 6.75 and 234.33. The number of average annual citations was 24.82. The median of the annual citations was 18.09 (11.82, 28.18). Five articles ranked in the top 10 in total citations were still ranked in the top 10 in annual citations, and most of them were recommendations or initiatives. The top 10 articles ranked in annual citations are shown in Table 1.

**Table 1:** The top 10 articles ranked by annual citations.

**Rank by** **NTC**	**Rank by** **NAC**	**Year of** **publication**	**Title**	**Number of** **total citations**	**Number of** **annual** **citations**	**First author**	**Corresponding** **author**	**Country** **of origin**	**Journal of publication**
5	1	2020	Asian Working Group for Sarcopenia: 2019 Consensus Update on Sarcopenia Diagnosis and Treatment	703	234.33	Chen, Liang-Kung	Chen, Liang-Kung;Arai, Hidenori;Woo, Jean	Taiwan	Journal of the American Medical Directions Association
3	2	2014	Prevalence of and interventions for sarcopenia in ageing adults: a systematic review. Report of the International Sarcopenia Initiative (EWGSOP and IWGS)	937	104.11	Cruz-Jentoft, Alfonso J.	Cruz-Jentoft, Alfonso J.	Spain	Age and Aging
2	3	2012	Lack of Exercise Is a Major Cause of Chronic Diseases	1,011	91.91	Booth, Frank W	Booth, Frank W	USA	Comprehensive Physiology
7	4	2014	Protein intake and exercise for optimal muscle function with aging: Recommendations from the ESPEN Expert Group	690	76.67	Deutz, Nicolaas E. P.	Deutz, Nicolaas E. P.	USA	Clinical Nutrition
41	5	2019	International Clinical Practice Guidelines for Sarcopenia (ICFSR): Screening, Diagnosis and Management	239	59.75	Dent, E.	Dent, E.	Australia	Journal of Nutrition Health & Aging
1	6	2003	Aging and sarcopenia	1,131	56.55	Timothy J. Doherty	Timothy J. Doherty	Canada	Journal of Applied Physiology
4	7	2009	Molecular inflammation: Underpinnings of aging and age-related diseases	783	55.93	Chung, Hae Young	Chung, Hae Young	South Korea	Aging Research Reviews
32	8	2018	Sarcopenic obesity in older adults: aetiology, epidemiology and treatment strategies	261	52.2	Batsis, John A	Batsis, John A	USA	Nature Reviews Endocrinology
57	9	2019	Resistance training for older adults: position statement from the national strength and conditioning association	205	51.25	Fragala, Maren S.	Fragala, Maren S.	USA	Journal of Strength and Conditioning Research
85	10	2019	Physical frailty: ICFSR international clinical practice guidelines for identification and management	172	43	Dent, E.	Dent, E.	Australia	Journal of Nutrition Health & Aging

**Figure 3:**
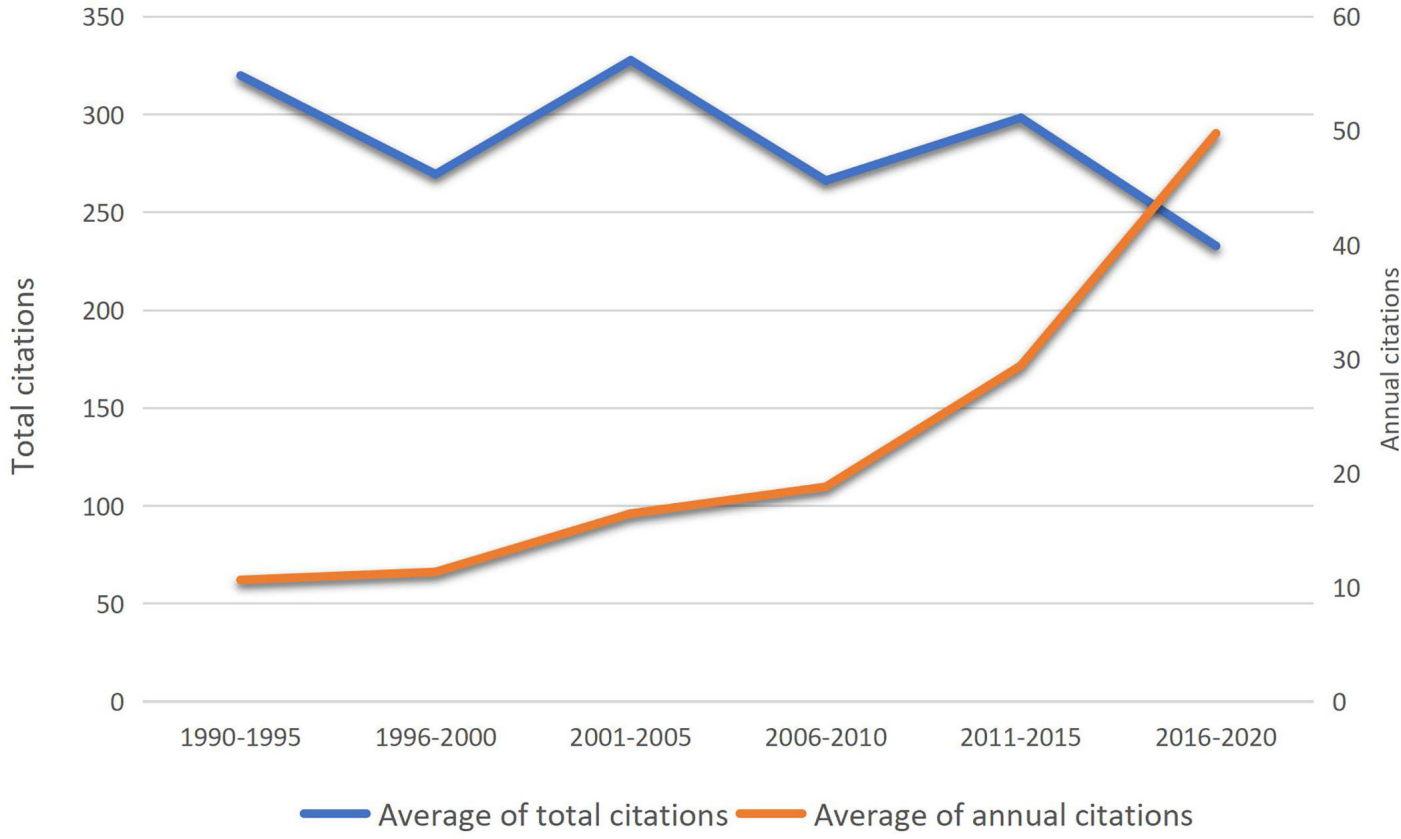
The average of total citations and annual citations in each 5-year interval.

The average of total citations and annual citations in each 5-year interval is shown in Figure 3. The average of annual citations was constantly increasing. No correlation exists between the total citations and annual citations (*r* = −0.655, *P* = 0.158).

### Article types and research focus

Among the 100 top-cited articles, 58 were original articles, and 42 were reviews. There was no significant difference in the median of total citations and annual citations between the review group and article group, 213.5 (183.25, 325) vs. 206 (183.25, 294.5; *P* = 0.494), 20.1 (13.66, 27.61) vs. 15.67 (11.1, 27.39; *P* = 0.780). Of the 58 original articles, 14 were randomized controlled trials (RCT), and 3 were meta-analyses.

Nineteen of the top 100 articles were comprehensive topics, such as diagnosis, epidemiology, pathological features, and interventions about sarcopenia; it means that interventions including exercise therapy are one of their discussion topics but not the only one significant topic. The rest of them (81%) were mainly focused on the interventions toward sarcopenia and could be classified into different research directions: effects of exercise therapy on physical performance (*n* = 44), cellular/molecular mechanisms (*n* = 35), and exercise as the prevention and predictor (*n* = 2).

**Figure 4:**
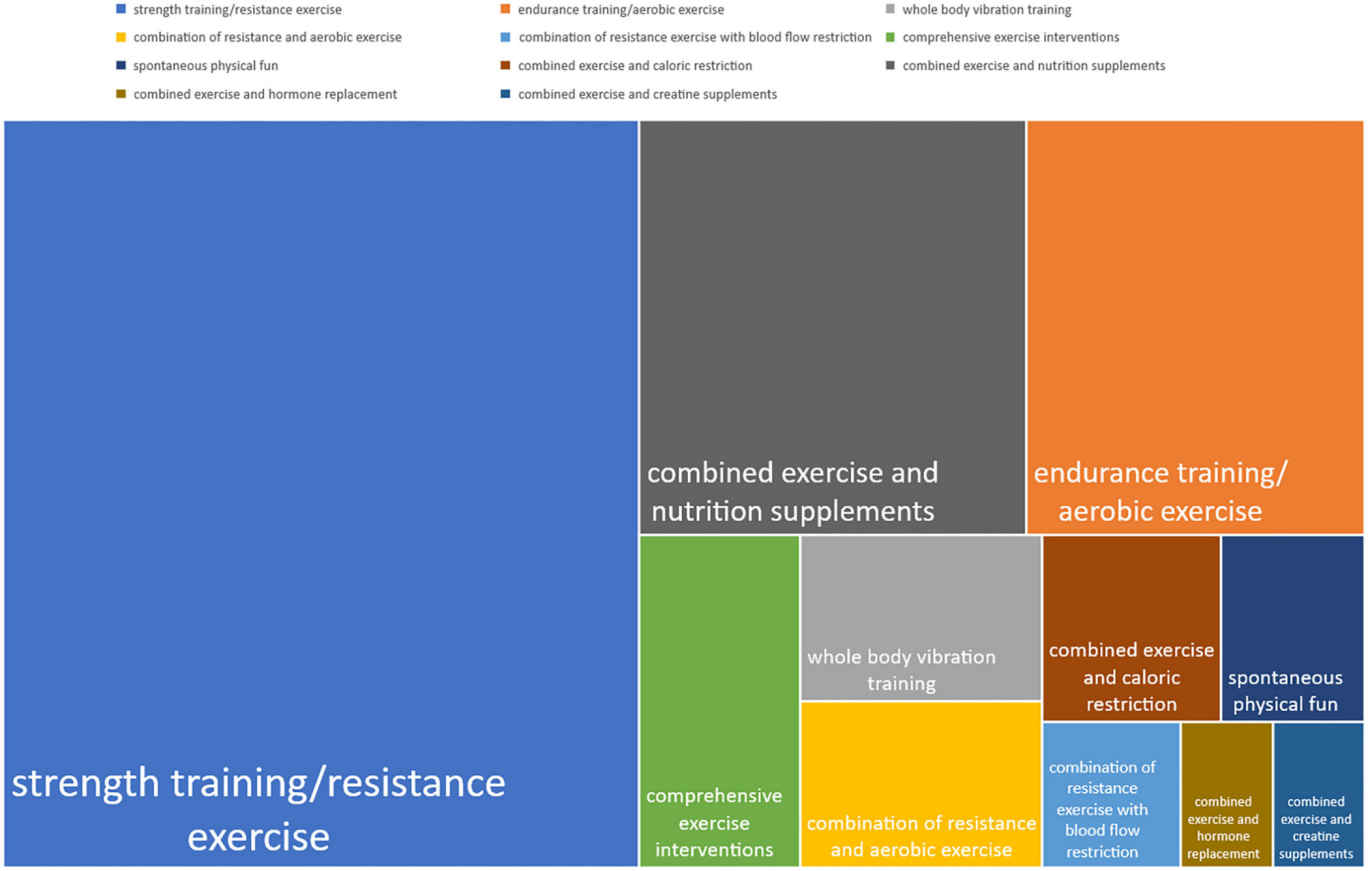
Different exercise strategies studied in the top 100 articles (size of the rectangle area represents frequency).

All the top 100 articles had studied or reviewed the use of exercise therapy in sarcopenia. Exercise interventions researched in the top 100 articles could be classified into different strategies (Figure 4): strength training/resistance exercise (71%), endurance training/aerobic exercise (21%), whole body vibration training (6%), combination of resistance and aerobic exercise (6%), combination of resistance exercise with blood flow restriction (3%), comprehensive exercise interventions (with different blends of aerobic, resistance, flexibility, and/or balance training) (8%), and spontaneous physical fun (SPF) (physical activities like brisk walking, dancing, yoga, and Tai Chi) (4%). Additionally, lots of articles researched or reviewed the combination of exercise with other therapies: nutrition supplements (24%), caloric restriction (5%), hormone replacement (2%), and creatine supplements (2%). Since many of the articles talked about more than one exercise strategy, the sum of percentages of exercise interventions included in these studies was greater than 1.

**Figure 5:**
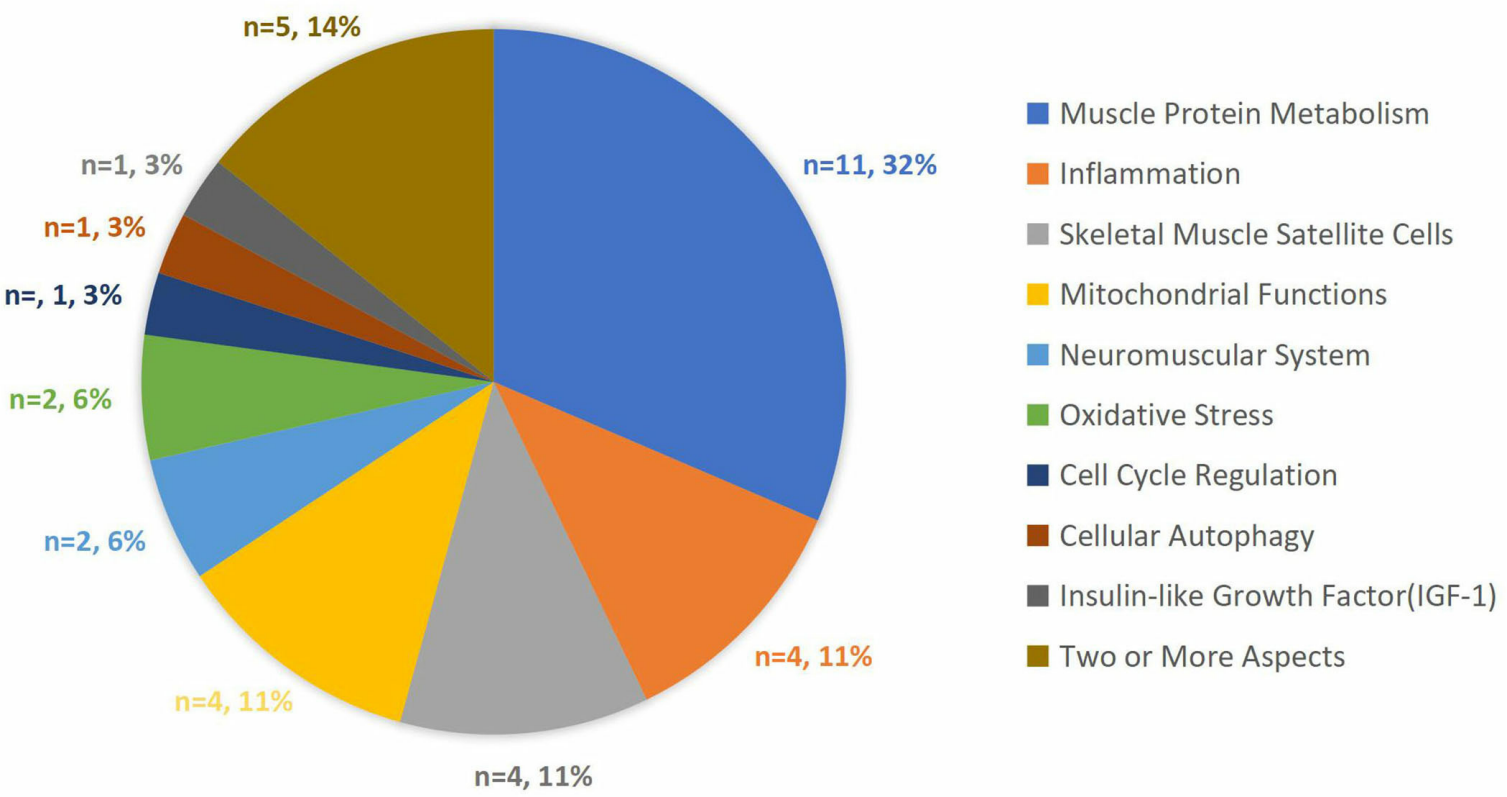
The proportion of the research orientations of the articles that focused on cellular/molecular mechanisms.

For articles focused on cellular/molecular mechanisms (*n* = 35), there were different research orientations: 11(31%) focused on muscle protein metabolism, while groups of articles focused on inflammation, skeletal muscle satellite cells, and mitochondrial functions, each containing 4(11%) articles, respectively; neuromuscular system and oxidative stress (OS) each containing 2 (6%) articles, one article on each of the three focuses on cell cycle regulation, cellular autophagy, and insulin-like growth factor (IGF-1); 5 (14%) articles focused on two or more aspects mentioned above (Figure 5). Four articles were using mice experiments to illustrate the mechanisms.

### Journals of publication

Fifty-four different journals made contributions to the list of top 100 articles. *Journal of Applied Physiology* was the journal that contributed the most articles (*n* = 8), followed by the *Journal of the American Medical Directors Association* (*n* = 7). *American Journal of Physiology-Endocrinology and Metabolism* and *Journals of Gerontology Series A-Biological Sciences and Medical Science* each contributed 5 and 4 articles, respectively. Among the 54 journals, 9 journals contributed more than or equal to 3 articles, 16 journals contributed 2 articles, and 29 journals contributed only 1 article. The impact factor and name of the journals that contributed more than or equal to 3 articles are shown in Table 2.

**Table 2:** Top-cited articles according to journal and their impact factors (only journals with 3 or more articles have been shown).

**Journal' name**	**Number of** **articles**	**2020 impact** **factor**
*Journal of Applied Physiology*	8	3.532
*Journal of the American Medical Directors Association*	7	4.669
*American Journal of Physiology-Endocrinology*	5	4.31
*Metabolism and Journals of Gerontology Series A-Biological Sciences and Medical Science*	4	6.053
*Age and Ageing*	3	10.668
*Ageing Research Reviews*	3	10.859
*Sports Medicine*	3	11.14
*Current Opinion in Clinical Nutrition and Metabolic Care*	3	4.294
*Journal of Nutrition Health & Aging*	3	4.075

### Countries, institutes, and corresponding authors

**Figure 6:**
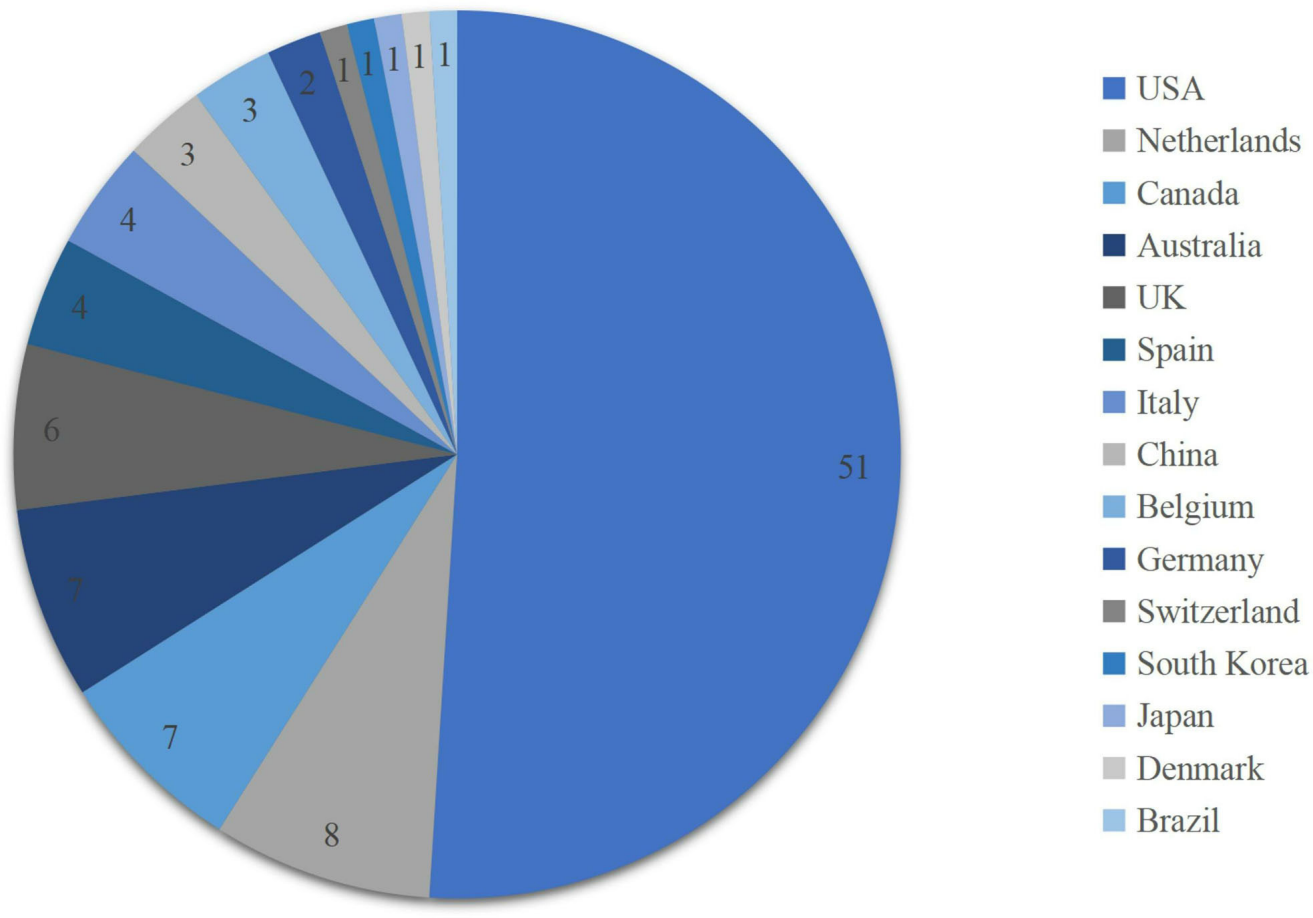
Original countries of the top 100 articles.

The top-cited 100 articles originated from 15 different countries [we regard Taiwan and the People's Republic of China as country China and regard England and Scotland as the United Kingdom (UK)], which are shown in Figure 6. The country of origin and the institute of research were identified based on the address of the first corresponding author. More than half of the articles were from the United States (*n* = 51), followed by Netherlands (*n* = 8), Canada (*n* = 7), Australia (*n* = 7), and the United Kingdom (*n* = 6). Considering the continents, authors from North America published the most (*n* = 58), followed by Europe (*n* = 29). Oceania and Asia each contributed 7 and 5 articles, respectively, and one of the articles was from Brazil, South America. None of the articles was from Africa.

We recorded the institute of the first corresponding author, and there were 8 institutes that contributed more than or equal to 3 articles (Table 3). Maastricht University published the most articles (*n* = 7), followed by Tufts University (*n* = 6) and University of Alabama at Birmingham (*n* = 5). University of Texas, University of Wisconsin, and Saint Louis University each published 4 articles, and McMaster University and University of Washington each published 3 articles.

**Table 3:** Institutes that contributed more or equal to three articles.

**Institute**	**Country**	**Number of** **articles**
Maastricht University	Netherlands	7
Tufts University	USA	6
University of Alabama at Birmingham	USA	5
University of Texas	USA	4
University of Wisconsin	USA	4
Saint Louis University	USA	4
McMaster University	Canada	3
University of Washington	USA	3

Among the top 100 articles, 4 corresponding authors published more than or equal to 3 articles. As a corresponding author, Luc J.C. van Loon from the Maastricht University in Netherlands published the most articles (*n* = 5), followed by Morley John E. from the Saint Louis University in the United States (*n* = 4), Marcas M. Bamman from the University of Alabama at Birmingham in the United States (*n* = 4), and Rasmussen, Blake B. from the University of Texas in the United States (*n* = 3).

## Discussion

On the issue of exercise therapy toward sarcopenia, strength training/resistance exercise has always been the most attention-getting intervention. In this article, we provided an overview of previous works concerning exercise therapy for sarcopenia, tremendously helping clinicians familiarize themselves with knowledge, as well as prospectively feasible strategies for this disease, research trends in this field, and then identify landmarks. To summarize, we found that the United States is the leader country in terms of contributions to development, and the increase in annual citations signifies that the issue of exercise therapy for sarcopenia is gradually attracting attention of more researchers.

Bibliometric analysis is a method to value the importance and influence of the publications from several aspects, thus providing specific and reliable data analysis on bibliometric parameters for researchers in certain fields by extracting and analyzing articles' characteristics. Citations of an article to some extent represent people's approbation level, and the top 100 most-cited articles might be the highly recognized articles in the area. By analyzing the top 100 articles' publication year, total and annual citations, impact factor and number of publication articles of the journal, types of literature and research focuses, as well as original countries, institutes, and corresponding authors, we could come to know their respective contributions in this field and different researching directions based on existing studies and thus may pave the way for the further studies.

From 1993 to 2020, the average of annual citations in each 5-year interval of the top 100 articles containing studies or reviews of exercise therapies for sarcopenia was continuously increasing, and the fastest-growing period is between the 2011–2015 interval and the 2016–2020 interval. It may attribute to articles published after 2012 among the top 20 articles ranked by annual citations were accounted for three quarters (*n* = 15). Since annual citations could to some extent eliminate the inherent bias caused by valuing an article by total citations, it may indicate that the quality of articles was increasing with years, and lots of high-quality articles were published during the recent decade. But there are two factors we cannot ignore; one factor is the obliteration by incorporation phenomenon (23, 24), which states that landmark articles are sometimes cited rarely because the information they provide becomes so widely used and embedded in the daily practice of each clinician and that researchers do not feel the need to cite that particular study. Obliteration by incorporation phenomenon sometimes leads to recent peak time periods of bibliometrics. The other factor is that people tend to cite newer articles as references.

We analyzed the article types and research contents in the top 100 articles; 19 of the top 100 articles were comprehensive topics. The rest of them (*n* = 81) were mainly focused on the interventions toward sarcopenia. Most of the articles (71%) discussed about strength training/resistance exercise, and most of them were practiced on arms or legs. Robbins et al. as the first corresponding authors successively published two articles about the lingual resistance exercise on treating dysphagia in older people, and both found a positive improvement in lingual muscle strength and mass after intervention (25, 26), which may enlighten us that the benefits of resistance exercise are applied to the whole body muscles. Among the 21 articles concerning endurance training/aerobic exercise, improvement in maximal oxygen consumption (VO2max) and mechanisms of systemic mitochondrial rejuvenation were mainly discussed. It follows that the two aspects are often essential when this kind of exercise functions according to studies. VO2max, able to serve as a major predictor of functional capacity, is the maximum capacity to transport and utilize oxygen and is often used as a measure of an individual's aerobic capacity (20, 27). Its level is related to muscle function and therefore can sensitively reflect the overall health status. Actually, VO2max is positively correlated with mitochondrial function and acts both as an indicator and a process that studies about endurance training/aerobic exercise usually focused on. Six articles discussed the combination of aerobic and resistance exercise, and among them, two RCT indicated that the combination of these two classic exercises could bring improvements both in muscle quality and physical capacity (28, 29). Although it was not clear whether the benefits of the intervention model of the combination of two exercises were simply equal to the sum of the benefits of a single exercise, we may recommend that patients with sarcopenia try to perform multiple exercises as much as the physical condition allows. Three articles of which two were meta-analyses discussed a relatively new strategy, resistance training combined blood flow restriction (BFR), and found a stronger improving effect than single resistance training; thus, the low-load resistance training combined BFR may provide a comparable surrogate for heavy-load training, which is of great significance for older people (30–32). Six articles discussed the effects of vibration therapy, and among them, three RCT found that whole-body-vibration training can effectively counteract the loss of muscle strength and mobility associated with age-induced sarcopenia (33–35). One of its remarkable advantages is the combined effect on the neuromuscular and neuroendocrine systems, and it could be a feasible treatment option for sarcopenia, as well as osteoporosis (36). Four articles discussed the SPF. As a series of popular daily activities suitable for all ages nowadays, SPF can not only prevent and treat physical diseases but also help people combat psychological ones (37). It is worth noting that, with an aging population worldwide, these activities could provide older adults relatively prone to suffer from mental illness with comprehensive help (38). Except for the pure exercise therapy, thirty-three articles in total explored the combination of exercise therapy with other therapies, including nutrition or creatine supplements, calorie restriction, and hormone replacement. For instance, Breen et al. (30) found that resistance exercise combined with amino acid ingestion results in the greatest anabolic response and may assist older adults in producing a “youthful” muscle protein synthetic response provided sufficient protein is ingested following exercise. More and more attention has been paid to multi-means comprehensive treatment.

There are thirty-five articles among the top 100 articles focused on the research of cellular/molecular mechanisms and details are shown in Figure 5. Reading these articles will help understand the pathophysiological process of sarcopenia and how exercise intervention works to combat or even reverse the underlying pathophysiology of sarcopenia in the microscopic field. The muscle protein metabolism, inflammation, and skeletal muscle satellite cells were the top 3 most-discussed topics among these thirty-five articles with a total of nineteen articles. Through them, we can learn much about the anabolic resistance of muscle protein synthesis in aging, the inflammation phenomenon like OS in aging, the potential role of exercise in the modulation of chronic inflammation, stem cells in aging, and how exercises influence the numbers of them. In addition, the mitochondrial function also deserved attention. Romanello et al. reviewed the changes in mitochondrial content, morphology and function in aging, and the beneficial effects of exercising in improving mitochondrial function by activating mitochondrial biogenesis and mitophagy. Moreover, there were other articles in this field talking about age-related attenuation in neuromuscular synapses, the decline of antioxidant defense ability with aging, cell cycle regulation, cellular autophagy, and insulin-like growth factor (IGF-1).

The top 100 most-cited articles were published in fifty-four different journals; among them, nine journals contributed more than or equal to three articles, and the maximum number of articles held by the same journal was 8. There was not an obvious centralized tendency about the articles' publication journal, which may be due to the various research directions and dynamic changes of study trends toward the exercise therapy for sarcopenia.

We identified 75 different first corresponding authors, and Luc J.C. van Loon published the most articles (*n* = 5). Most of the authors (77.3%) published only one article; 87% of articles originated from North America (58%) and Europe (29%); and more than half of the articles originated from the United States (51%). These findings that most of the influential articles came from North American countries, especially the United States, and European countries were consistent with bibliometric analyses in other fields, such as a certain drug or protein, nervous system diseases, and musculoskeletal system diseases similar to ours (39–42). Different degrees of economy and technology level might explain this result, and regarding our study, it might also partly attribute to the higher prevalence of sarcopenia in those areas (43).

There are several limitations in our study. First, although the number of total citations is an important indicator of the quality and attractiveness of an article, a certain amount of time is needed for an article to accumulate citations after publishing. For this reason, using only the number of citations is inadequate for determining the value of an article and may lead to the omittance of some high-quality but relatively newer articles. Second, this citation analysis did not exclude the influence of self-citations, citations in lectures or conferences, and authors' potential preference to cite articles from a specific journal, which may introduce bias in our results (44, 45). Third, articles were only retrieved from one database. There are several other databases, including Cochrane Library, Embase, PubMed, allowing researchers to access influential works, which this article ignored, indicating potential omissions. Even some publications might not be available in all these search engines. Furthermore, one author would present different affiliations in some articles in a few cases due to his different periods of academic career, which may bring bias in contributing countries. Moreover, we included articles that studied or reviewed the use of exercise therapy in sarcopenia, valuing and extracting them by their total citations. Since several articles in our top 100 list were not only containing exercise therapy for sarcopenia, especially articles that discussed the comprehensive topics of sarcopenia, we could not exclude the possibilities that their higher total citations may partly attribute to the other bright spots of contents other than exercise intervention. Last but not least, the pronounced limitations of our study are inherent to citation analysis. The total citations of one article could be influenced by the journal level, and an article published in a journal with a great impact factor could be more likely to receive more total citations (46). In addition, we cannot deny the existence of the phenomenon, termed “obliteration by incorporation”, that some insightful articles were cited a limited number of times until their findings became well known (46). In a word, highly cited publications are not fully equal to good quality because they may be affected by multiple factors except for content, such as article age, author's reputation, institution, and original language.

However, the inherent strengths of this study are remarkable despite the limitations mentioned above. Bibliometric analysis is a statistical evaluation of publications, and it could provide relatively comprehensive data information of the published articles for physicians and researchers in a certain field (44, 47, 48). Although it is virtually impossible to evaluate the true value of an article, citation analysis provides a simple quantitative technique to estimate the impact of an article since the articles owing high citations may be highly recognized in this field. Thus, bibliometric analysis based on citation frequency remains the most commonly used tool at present in order to identify important discoveries and studies that have had a disproportionate influence in a particular field (23, 49).

## Conclusion

Our study provides a detailed bibliometric analysis about the top 100 most-cited articles on exercise therapy for sarcopenia. We observed a strong ascending trend for the number of annual citations, and it may be a hint that people's interest and attention in this field are increasing. Most articles focused on strength/resistance training, endurance/aerobic training, and exercise combined nutrition supplement treatments. The optimal strategy is still being explored, and some novel interventions like blood flow restriction training and whole-body-vibration training seem to be feasible and potential. The illustration of optimal strategy may rely upon the research of cellular/molecular mechanisms caused by exercise treatments. Contributions of journals, authors, institutes, and original countries were analyzed in this study. In a word, this study determined the top 100 most-cited articles on exercise therapy for sarcopenia and analyzed their bibliometric characteristics, which may provide a recommended list for researchers in this field and pave the way for further study.

## Data availability statement

The raw data supporting the conclusions of this article will be made available by the authors, without undue reservation.

## Author contributions

Z-jG and W-qX had the original idea and wrote the first draft. W-qX helped Z-jG make decisions when it comes to confusing situations. Z-jG, SN, and Z-jC revised the manuscript. Y-yZ and Y-lD oversaw the search and conducted the title/abstract review and analysis with assistance from Z-jG and W-qX. Y-sL and W-fX brainstormed on the topic and revised the manuscript. All authors contributed to the article and approved the submitted version.

## Funding

This study was supported by the National Key R&D Program of China (No. 2019YFA0111900), the National Natural Science Foundation of China (No. 81874030 and 82072506), Provincial Clinical Medical Technology Innovation Project of Hunan (No. 2020SK53709), the Administration of Traditional Chinese Medicine of Hunan Province (No. 2021075), the Innovation-Driven Project of Central South University (No. 2020CX045), the Wu Jieping Medical Foundation (No. 320.6750.2020-03-14), the Key Program of Health Commission of Hunan Province (No. 20201902), the Independent Exploration and Innovation Project for Postgraduate Students of Central South University (No. 2021zzts1024 and No. 2021zzts1030), National Clinical Research Center for Geriatric Disorders (No. 2021KFJJ02 and 2021LNJJ05), National Clinical Research Center for Orthopedics, Sports Medicine and Rehabilitation (No. 2021-NCRC-CXJJ-PY-40), Provincial Outstanding Postdoctoral Innovative Talents Program (No. 2021RC2020), and the Hunan Provincial Innovation Foundation for Postgraduate (No. CX20210360).

## Conflict of interest

The authors declare that the research was conducted in the absence of any commercial or financial relationships that could be construed as a potential conflict of interest.

## Publisher's note

All claims expressed in this article are solely those of the authors and do not necessarily represent those of their affiliated organizations, or those of the publisher, the editors and the reviewers. Any product that may be evaluated in this article, or claim that may be made by its manufacturer, is not guaranteed or endorsed by the publisher.
