# Supplementary material for: The top 100 most-cited articles on exercise therapy for sarcopenia: A bibliometric analysis
Source: Front Med (Lausanne). 2022 Aug 12;9:961318. doi: 10.3389/fmed.2022.961318 (PMC9412013; doi:10.3389/fmed.2022.961318)
Supplement: Supplementary file 1 [file Table_1.DOCX]

**Supplement Table 1.** List of the top 100 most-cited articles on exercise therapy for sarcopenia

| **Rank by NTC** | **Rank by NAC** | **Year of Publication** | **Title** | **Number of Total Citations** | **Number of Annual Citations** | **First Author** | **Corresponding Author** | **Country of Origin** | **Journal of Publication** |
| --- | --- | --- | --- | --- | --- | --- | --- | --- | --- |
| 1 | 6 | 2003 | Aging and sarcopenia | 1131 | 56.55 | Timothy J. Doherty | Timothy J. Doherty | Canada | Journal of Applied Physiology |
| 2 | 3 | 2012 | Lack of Exercise Is a Major Cause of Chronic Diseases | 1011 | 91.91 | Booth, Frank W | Booth, Frank W | USA | Comprehensive Physiology |
| 3 | 2 | 2014 | Prevalence of and interventions for sarcopenia in ageing adults: a systematic review. Report of the International Sarcopenia Initiative (EWGSOP and IWGS) | 937 | 104.11 | Cruz-Jentoft, Alfonso J. | Cruz-Jentoft, Alfonso J. | Spain | Age and Ageing |
| 4 | 7 | 2009 | Molecular inflammation: Underpinnings of aging and age-related diseases | 783 | 55.93 | Chung, Hae Young | Chung, Hae Young | South Korea | Ageing Research Reviews |
| 5 | 1 | 2020 | Asian Working Group for Sarcopenia: 2019 Consensus Update on Sarcopenia Diagnosis and Treatment | 703 | 234.33 | Chen, Liang-Kung | Chen, Liang-Kung；Arai, Hidenori；Woo, Jean | Taiwan | Journal of the American Medical Directions Association |
| 6 | 18 | 2001 | Sarcopenia | 693 | 31.5 | Morley, JE | Morley, JE | USA | Journal of Laboratory and Clinical Medicine |
| 7 | 4 | 2014 | Protein intake and exercise for optimal muscle function with aging: Recommendations from the ESPEN Expert Group | 690 | 76.67 | Deutz, Nicolaas E. P. | Deutz, Nicolaas E. P. | USA | Clinical Nutrition |
| 8 | 37 | 1999 | Predictors of skeletal muscle mass in elderly men and women | 559 | 23.29 | Baumgartner, RN | Morley, JE | USA | Mechanisms of Ageing and Development |
| 9 | 33 | 2002 | Aging of the human neuromuscular system | 518 | 24.67 | Vandervoort, AA | Vandervoort, AA | Canada | Muscle & Nerve |
| 10 | 44 | 2000 | Sarcopenia: Current concepts | 447 | 19.43 | Roubenoff, R | Roubenoff, R | USA | Journals of Gerontology Series A-biological Science and Medical Sciences |
| 11 | 39 | 2004 | Effects of resistance training on older adults | 431 | 22.68 | Hunter, GR | Hunter, GR | USA | Sports Medicine |
| 12 | 16 | 2010 | Combined Resistance and Aerobic Exercise Program Reverses Muscle Loss in Men Undergoing Androgen Suppression Therapy for Prostate Cancer Without Bone Metastases: A Randomized Controlled Trial | 416 | 32 | Galvao, Daniel A. | Galvao, Daniel A. | Australia | Journal of Clinical Oncology |
| 13 | 19 | 2010 | Role of the nervous system in sarcopenia and muscle atrophy with aging: strength training as a countermeasure | 409 | 31.46 | Aagaard, P. | Aagaard, P. | Denmark | Scandinavian Journal of Medicine & Science in Sports |
| 14 | 13 | 2012 | Protein Supplementation Increases Muscle Mass Gain During Prolonged Resistance-Type Exercise Training in Frail Elderly People: A Randomized, Double-Blind, Placebo-Controlled Trial | 391 | 35.55 | Tieland, Michael | Tieland, Michael | Netherlands | Journal of the American Medical Directions Association |
| 15 | 22 | 2010 | Nutritional Recommendations for the Management of Sarcopenia | 391 | 30.08 | Morley, John E. | Morley, John E | USA | Journal of the American Medical Directions Association |
| 16 | 24 | 2010 | Resistance exercise for muscular strength in older adults: A meta-analysis | 367 | 28.23 | Peterson, Mark D. | Peterson, Mark D. | USA | Ageing Research Reviews |
| 17 | 73 | 1993 | Sarcopenia and Age-Related Changes in Body Composition and Functional Capacity | 366 | 12.2 | William J. Evans | William J. Evans | USA | The Journal of Nutrition |
| 18 | 51 | 2004 | Muscle strength, power and adaptations to resistance training in older people | 342 | 18 | Macaluso, A | Macaluso, A | Scotland | European Journal of Applied Physiology |
| 19 | 62 | 2001 | Resistance exercise decreases skeletal muscle tumor necrosis factor alpha in frail elderly humans | 333 | 15.14 | Greiwe, JS | Semenkovich, CF | USA | Faseb Journal |
| 20 | 26 | 2011 | Influence of Resistance Exercise on Lean Body Mass in Aging Adults: A Meta-Analysis | 331 | 27.58 | Peterson, Mark D. | Peterson, Mark D. | USA | Medicine & Science in Sports & Exercise |
| 21 | 15 | 2013 | The decline in skeletal muscle mass with aging is mainly attributed to a reduction in type II muscle fiber size | 326 | 32.6 | Nilwik, Rachel | van Loon, Luc J. C. | Netherlands | Experimental Gerontology |
| 22 | 34 | 2010 | Attenuation of age-related changes in mouse neuromuscular synapses by caloric restriction and exercise | 318 | 24.46 | Valdez, Gregorio | Lichtman, Jeff W. | USA | Proceedings of the National Academy of Science of the United States of America |
| 23 | 70 | 1999 | Effects of heavy-resistance training on hormonal response patterns in younger vs. older men | 313 | 13.04 | Kraemer, WJ | Kraemer, WJ | USA | Journal of Applied Physiology |
| 24 | 25 | 2012 | Resistance exercise enhances myofibrillar protein synthesis with graded intakes of whey protein in older men | 310 | 28.18 | Yang, Yifan | Phillips, Stuart M. | Canada | British Journal of Nutrition |
| 25 | 50 | 2006 | Effificacy of 3 days/wk resistance training on myofifiber hypertrophy and myogenic mechanisms in young vs. older adults | 309 | 18.18 | Kosek, David J | Bamman, Marcas M. | USA | Journal of Applied Physiology |
| 26 | 27 | 2012 | Effects of Exercise and Amino Acid Supplementation on Body Composition and Physical Function in Community-Dwelling Elderly Japanese Sarcopenic Women: A Randomized Controlled Trial | 295 | 26.82 | Kim, Hun Kyung | Kim, Hun Kyung | Japen | Journal of the American Geriatrics Society |
| 27 | 40 | 2010 | Skeletal muscle autophagy and apoptosis during aging: Effects of calorie restriction and life-long exercise | 293 | 22.54 | Wohlgemuth, Stephanie Eva | Wohlgemuth, Stephanie Eva | USA | Experimental Gerontology |
| 28 | 59 | 2005 | The effects of lingual exercise on swallowing in older adults | 285 | 15.83 | Robbins, J | Robbins, J | USA | Journal of the American Geriatrics Society |
| 29 | 91 | 1993 | Changes in skeletal muscle with aging: effects of exercise training | 274 | 9.13 | Rogers, M A | William J. Evans | USA | Exercise and Sport Sciences Reviews |
| 30 | 38 | 2011 | Skeletal muscle protein metabolism in the elderly: Interventions to counteract the 'anabolic resistance' of ageing | 273 | 22.75 | Breen, Leigh | Phillips, Stuart M. | Canada | Nutrition & Metabolism |
| 31 | 42 | 2010 | Oxidative Stress, Molecular Inflammation and Sarcopenia | 270 | 20.77 | Meng, Si-Jin | Yu, Long-Jiang | Peoples R China | International Journal of Molecular Science |
| 32 | 8 | 2018 | Sarcopenic obesity in older adults: aetiology, epidemiology and treatment strategies | 261 | 52.2 | Batsis, John A | Batsis, John A | USA | Nature Reviews Endocrinology |
| 33 | 66 | 2005 | Whole body vibration exercise: are vibrations good for you? | 260 | 14.44 | Cardinale, M | Cardinale, M | Scotland | British Journal of Nutrition |
| 34 | 43 | 2010 | Protecting muscle mass and function in older adults during bed rest | 260 | 20 | English, Kirk L | Paddon-Jones, Douglas | USA | Current Opinion in Clinical Nutrition and Metabolic Care |
| 35 | 55 | 2008 | Skeletal muscle protein anabolic response to resistance exercise and essential amino acids is delayed with aging | 259 | 17.27 | Drummond, Micah J. | Rasmussen, Blake B. | USA | Journal of Applied Physiology |
| 36 | 56 | 2008 | Sarcopenia: Diagnosis and treatment | 252 | 16.8 | Morley, J. E. | Morley, J. E. | USA | Journal of Nutrition Health & Aging |
| 37 | 81 | 1999 | Muscle quality. II. Effects of strength training in 65- to 75-yr-old men and women | 249 | 10.83 | Tracy, BL | Hurley, BF | USA | Journal of Applied Physiology |
| 38 | 82 | 2000 | Sarcopenia and its implications for the elderly | 248 | 10.78 | Roubenoff, R | Roubenoff, R | USA | European Journal of Clinical Nutrition |
| 39 | 67 | 2006 | Efficacy of myonuclear addition may explain differential myofiber growth among resistance-trained young and older men and women | 245 | 14.41 | Petrella, John K. | Bamman, Marcas M. | USA | American Journal of Physiology-Endocrinology and Metabolism |
| 40 | 77 | 2002 | Exercise-induced modulation of antioxidant Defense | 241 | 11.48 | Ji, LL | Ji, LL | USA | Increasing Healthy Life Span: Conventional Measures and Slowing the Innate Aging Process |
| 41 | 5 | 2019 | International Clinical Practice Guidelines for Sarcopenia (ICFSR): Screening, Diagnosis and Management | 239 | 59.75 | Dent, E. | Dent, E. | Australia | Journal of Nutrition Health & Aging |
| 42 | 41 | 2012 | Chronic low-grade inflammation and age-related sarcopenia | 236 | 21.45 | Beyer, Ingo | Bautmans, Ivan | Belgium | Current Opinion in Clinical Nutrition and Metabolic Care |
| 43 | 11 | 2017 | Aerobic or Resistance Exercise, or Both, in Dieting Obese Older Adults | 235 | 39.17 | Villareal, Dennis T | Villareal, Dennis T | USA | New England Journal of Medicine |
| 44 | 45 | 2011 | Endurance exercise rescues progeroid aging and induces systemic mitochondrial rejuvenation in mtDNA mutator mice | 233 | 19.42 | Safdar, Adeel | Tarnopolsky, Mark A. | Canada | Proceedings of the National Academy of Science of the United States of America |
| 45 | 57 | 2009 | Aging, exercise, and muscle protein metabolism | 233 | 16.64 | Koopman, Rene | van Loon, Luc J. C. | Netherlands | Journal of Applied Physiology |
| 46 | 54 | 2010 | A systematic review of the separate and combined effects of energy restriction and exercise on fat-free mass in middle-aged and older adults: implications for sarcopenic obesity | 225 | 17.31 | Weinheimer, Eileen M | Campbell, Wayne W | USA | Nutrition Reviews |
| 47 | 48 | 2011 | Aging impairs contraction-induced human skeletal muscle mTORC1 signaling and protein synthesis | 224 | 18.67 | Fry, Christopher S. | Rasmussen, Blake B. | USA | Skeletal Muscle |
| 48 | 76 | 2004 | Interventions for sarcopenia and muscle weakness in older people | 223 | 11.74 | Borst, SE | Borst, SE | USA | Age and Ageing |
| 49 | 12 | 2017 | Nutrition and physical activity in the prevention and treatment of sarcopenia: systematic review | 215 | 35.83 | Beaudart, C. | Cooper, C. | England | Osteoporosis International |
| 50 | 68 | 2008 | Efficacy of progressive resistance training on balance performance in older adults: A systematic review Randomized controlled trials | 212 | 14.13 | Orr, Rhonda | Orr, Rhonda | Australia | Sports Medicine |
| 51 | 21 | 2016 | Mitochondrial Quality Control and Muscle Mass Maintenance | 211 | 30.14 | Romanello, Vanina | Romanello, Vanina | Italy | Frontiers in Physiology |
| 52 | 35 | 2014 | Sarcopenia, Cachexia and Aging: Diagnosis, Mechanisms and Therapeutic Options - A Mini-Review | 211 | 23.44 | Ali, Sumbul | Garcia, Jose M | USA | Gerontology |
| 53 | 64 | 2009 | Changes in muscle mass and strength after menopause | 209 | 14.93 | Maltais, M. L. | Dionne, I. J. | Canada | Journal of Musculoskeletal & Neuronal Interactions |
| 54 | 92 | 2000 | Strength training in the elderly - Effects on risk factors for age-related diseases | 207 | 9 | Hurley, BF | Hurley, BF | USA | Sports Medicine |
| 55 | 96 | 1997 | Departures from linearity in the relationship between measures of muscular strength and physical performance of the lower extremities: the Women's Health and Aging Study. | 207 | 7.96 | Ferrucci, L | Ferrucci, L | Italy | Journals of Gerontology Series A-biological Sciences and Medical Science |
| 56 | 29 | 2015 | Skeletal muscle wasting in cachexia and sarcopenia: molecular pathophysiology and impact of exercise training | 206 | 25.75 | Bowen, T. Scott | Adams, Volker | Germany | Journal of Cachexia Sarcopenia and Muscle |
| 57 | 9 | 2019 | Resistance Training for Older Adults: Position Statement From the National Strength and Conditioning Association | 205 | 51.25 | Fragala, Maren S. | Fragala, Maren S. | USA | Journal of Strength and Conditioning Research |
| 58 | 72 | 2007 | Resistance Exercise Reverses Aging in Human Skeletal Muscle | 204 | 12.75 | Melov, Simon | Melov, Simon | USA | Plos One |
| 59 | 93 | 2000 | Resistance exercise acutely increases MHC and mixed muscle protein synthesis rates in 78-84 and 23-32 yr olds | 204 | 8.87 | Hasten, DL | Yarasheski, KE | USA | American Journal of Physiology-Endocrinology and Metabolism |
| 60 | 65 | 2009 | Aerobic exercise training improves whole muscle and single myofiber size and function in older women | 203 | 14.5 | Harber, Matthew P. | Harber, Matthew P. | USA | American Journal of Physiology-Regulatory Integrative and Comparative Physiology |
| 61 | 94 | 2009 | Senescent Swallowing: Impact, Strategies, and Interventions | 203 | 8.46 | Ney, Denise M. | Robbins, JoAnne | USA | Nutrition in Clinical Practice |
| 62 | 75 | 2006 | Resistance training and reduction of treatment side effects in prostate cancer patients | 201 | 11.82 | Galvao, Daniel A. | Galvao, Daniel A. | Australia | Medicine and Science in Sports and Exercise |
| 63 | 61 | 2010 | Blood flow restriction exercise stimulates mTORC1 signaling and muscle protein synthesis in older men | 200 | 15.38 | Fry, Christopher S. | Rasmussen, Blake B. | USA | Journal of Applied Physiology |
| 64 | 14 | 2017 | The Role of Inflammation in Age-Related Sarcopenia | 198 | 33 | Dalle, Sebastiaan | Koppo, Katrien | Belgium | Frontiers in Physiology |
| 65 | 85 | 2003 | The benefits of strength training for older adults | 198 | 9.9 | Seguin, R | Nelson, ME | USA | American Journal of Preventive Medicine |
| 66 | 86 | 2003 | The positive effects of negative work: Increased muscle strength and decreased fall risk in a frail elderly population | 197 | 9.85 | LaStayo, PC | LaStayo, PC | USA | Journals of Gerontology Series A-biological Sciences and Medical Science |
| 67 | 58 | 2011 | Exercising before protein intake allows for greater use of dietary protein-derived amino acids for de novo muscle protein synthesis in both young and elderly men | 196 | 16.33 | Pennings, Bart | van Loon, Luc J. C. | Netherlands | American Journal of Clinical Nutrition |
| 68 | 90 | 2012 | Effects of High-Intensity Progressive Resistance Training and Targeted Multidisciplinary Treatment of Frailty on Mortality and Nursing Home Admissions after Hip Fracture: A Randomized Controlled Trial | 192 | 9.14 | Singh, Nalin A. | Singh, Maria A. Fiatarone | Australia | Journal of the American Medical Directions Association |
| 69 | 88 | 2003 | Sarcopenia-consequences, mechanisms; and potential therapies | 188 | 9.4 | Greenlund, LJS | Nair, KS | USA | Mechanisms of Ageing and Development |
| 70 | 20 | 2017 | Blood flow restriction training in clinical musculoskeletal rehabilitation: a systematic review and meta-analysis | 187 | 31.17 | Hughes, Luke | Hughes, Luke | England | British Journal of Sports Medicine |
| 71 | 28 | 2016 | Whey protein, amino acids, and vitamin D supplementation with physical activity increases fat-free mass and strength, functionality, and quality of life and decreases inflammation in sarcopenic elderly | 187 | 26.71 | Rondanelli, Mariangela | Rondanelli, Mariangela | Italy | American Journal of Clinical Nutrition |
| 72 | 36 | 2015 | Prevention and optimal management of sarcopenia: a review of combined exercise and nutrition interventions to improve muscle outcomes in older people | 187 | 23.38 | Denison, Hayley J. | Sayer, Avan Aihie | England | Clinical Interventions in Aging |
| 73 | 98 | 1999 | Insulin-like growth factor I in skeletal muscle after weight-lifting exercise in frail elders | 187 | 7.79 | Singh, MAF | Singh, MAF | USA | American Journal of Physiology-Endocrinology and Metabolism |
| 74 | 84 | 2005 | Sarcopenia of aging and its metabolic impact | 184 | 10.22 | Karakelides, H | Karakelides, H | USA | Current Topics in Developmental Biology, Volume 68 |
| 75 | 49 | 2013 | Anabolic Resistance of Muscle Protein Synthesis with Aging | 183 | 18.3 | Burd, Nicholas A. | van Loon, Luc J. C. | Netherlands | Exercise and Sports Sciences Reviews |
| 76 | 99 | 1998 | Impact of resistance exercise during bed rest on skeletal muscle sarcopenia and myosin isoform distribution. | 182 | 7.28 | Bamman, M M | Bamman, M M | USA | Journal of Applied Physiology |
| 77 | 83 | 2006 | Satellite cell numbers in young and older men 24 hours after eccentric exercise | 178 | 10.47 | Dreyer, HC | Dreyer, HC | USA | Muscle & Nerve |
| 78 | 30 | 2014 | Satellite cells in human skeletal muscle; from birth to old age | 177 | 25.26 | Verdijk, Lex B | Verdijk, Lex B | Netherlands | Age |
| 79 | 53 | 2013 | Role of exercise on sarcopenia in the elderly | 177 | 17.7 | Montero-Fernandez, N. | Montero-Fernandez, N. | Spain | European Journal of Physical and Rehabilitation Medicine |
| 80 | 80 | 2007 | Impact of whole-body vibration training versus fitness training on muscle strength and muscle mass in older men: A 1-year randomized controlled trial | 177 | 11.06 | Bogaerts, An | Delecluse, Christophe | Belgium | Journals of Gerontology Series A-biological Sciences and Medical Science |
| 81 | 31 | 2016 | Recent Advances in Sarcopenia Research in Asia: 2016 Update From the Asian Working Group for Sarcopenia | 176 | 25.14 | Chen, Liang-Kung | Chen, Liang-Kung | Taiwan | Journal of the American Medical Directions Association |
| 82 | 87 | 2005 | Impact of resistance loading on myostatin expression and cell cycle regulation in young and older men and women | 176 | 9.78 | Kim, JS | Bamman, MM | USA | American Journal of Physiology-Endocrinology and Metabolism |
| 83 | 89 | 2004 | Muscle tissue changes with aging | 176 | 9.23 | Volpi, E | Volpi, E | USA | Current Opinion in Clinical Nutrition and Metabolic Care |
| 84 | 32 | 2016 | Skeletal Muscle Regulates Metabolism via Interorgan Crosstalk: Roles in Health and Disease | 173 | 24.71 | Argiles, Josep M. | Argiles, Josep M. | Spain | Journal of the American Medical Directions Association |
| 85 | 10 | 2019 | Physical Frailty: ICFSR International Clinical Practice Guidelines for Identification and Management | 172 | 43 | Dent, E. | Dent, E. | Australia | Journal of Nutrition Health & Aging |
| 86 | 23 | 2017 | Osteosarcopenia: where bone, muscle, and fat collide | 172 | 28.67 | Hirschfeld, H. P. | Duque, G. | Brazil | Osteoporosis International |
| 87 | 47 | 2014 | The neuromuscular junction: aging at the crossroad between nerves and muscle | 170 | 18.89 | Gonzalez-Freire, Marta | Gonzalez-Freire, Marta | USA | Frontiers in Aging Neuroscience |
| 88 | 71 | 2010 | Skeletal muscle wasting in cachexia and sarcopenia: molecular pathophysiology and impact of exercise training | 168 | 12.92 | Lenk, Karsten | Adams, Volker | Germany | Journal of Cachexia Sarcopenia and Muscle |
| 89 | 95 | 2003 | High-frequency vibration training increases muscle power in postmenopausal women | 168 | 8.4 | Russo, CR | Ferrucci, L | USA | Archives of Physical Medicine and Rehabilitation |
| 90 | 63 | 2012 | Skeletal muscle mitochondria and aging: a review. | 166 | 15.09 | Peterson, Courtney M | Darcy L. Johannsen | USA | Journal of Aging Research |
| 91 | 52 | 2014 | The role of dietary protein and vitamin D in maintaining musculoskeletal health in postmenopausal women: A consensus statement from the European Society for Clinical and Economic Aspects of Osteoporosis and Osteoarthritis (ESCEO) | 162 | 18 | Rizzoli, Rene | Rizzoli, Rene | Switzerland | Maturitas |
| 92 | 69 | 2011 | Menopause and sarcopenia: A potential role for sex hormones | 162 | 13.5 | Messier, Virginie | Aubertin-Leheudre, Mylene | Canada | Maturitas |
| 93 | 100 | 1999 | Resistance exercise training increases mixed muscle protein synthesis rate in frail women and men >= 76 yr old | 162 | 6.75 | Yarasheski, KE | Yarasheski, KE | USA | American Journal of Physiology-Endocrinology and Metabolism |
| 94 | 17 | 2018 | Oxidative stress: role of physical exercise and antioxidant nutraceuticals in adulthood and aging | 159 | 31.8 | Simioni, Carolina | Luca M. Neri, | Italy | Oncotarget |
| 95 | 78 | 2009 | The impact of sarcopenia and exercise training on skeletal muscle satellite cells | 159 | 11.36 | Snijders, Tim | van Loon, Luc. J. C. | Netherlands | Ageing Research Reviews |
| 96 | 60 | 2013 | New horizons in the pathogenesis, diagnosis and management of sarcopenia | 157 | 15.7 | Sayer, Avan Aihie | Sayer, Avan Aihie | England | Age and Ageing |
| 97 | 79 | 2009 | Alterations in Muscle Attenuation following Detraining and Retraining in Resistance-Trained Older Adults | 157 | 11.21 | Taaffe, Dennis R. | Taaffe, Dennis R. | Australia | Gerontology |
| 98 | 97 | 2003 | Sarcopenia and aging | 156 | 7.8 | Kamel, HK | Kamel, HK | USA | Nutrition Reviews |
| 99 | 46 | 2015 | There Are No Nonresponders to Resistance-Type Exercise Training in Older Men and Women | 155 | 19.38 | Churchward-Venne, Tyler A. | van Loon, Luc J. C. | Netherlands | Journal of the American Medical Directors Association |
| 100 | 74 | 2010 | Whole-body vibration training increases muscle strength and mass in older women: a randomized-controlled trial | 155 | 11.92 | Machado, A. | Garatachea, N. | Spain | Scandinavian Journal of Medicine & Science in Sports |
